# m^6^A Regulates Neurogenesis and Neuronal Development by Modulating Histone Methyltransferase Ezh2

**DOI:** 10.1016/j.gpb.2018.12.007

**Published:** 2019-05-30

**Authors:** Junchen Chen, Yi-Chang Zhang, Chunmin Huang, Hui Shen, Baofa Sun, Xuejun Cheng, Yu-Jie Zhang, Yun-Gui Yang, Qiang Shu, Ying Yang, Xuekun Li

**Affiliations:** 1 The Children's Hospital, School of Medicine, Zhejiang University, Hangzhou 310052, China; 2 The Institute of Translational Medicine, School of Medicine, Zhejiang University, Hangzhou 310029, China; 3 CAS Key Laboratory of Genomic and Precision Medicine, Collaborative Innovation Center of Genetics and Development, College of Future Technology, Beijing Institute of Genomics, Chinese Academy of Sciences, Beijing 100101, China; 4 University of Chinese Academy of Sciences, Beijing 100049, China; 5 Sino-Danish College, University of Chinese Academy of Sciences, Beijing 101408, China; 6 Institute of Stem Cell and Regeneration, Chinese Academy of Sciences, Beijing 100101, China

**Keywords:** *N*^6^-methyladenosine (m^6^A), Mettl3, Neurogenesis, Neuronal development, *Ezh2*

## Abstract

***N*^6^-methyladenosine (m^6^A)**, catalyzed by the methyltransferase complex consisting of **Mettl3** and Mettl14, is the most abundant RNA modification in mRNAs and participates in diverse biological processes. However, the roles and precise mechanisms of m^6^A modification in regulating **neuronal development** and adult **neurogenesis** remain unclear. Here, we examined the function of Mettl3, the key component of the complex, in neuronal development and adult neurogenesis of mice. We found that the depletion of *Mettl3* significantly reduced m^6^A levels in adult neural stem cells (aNSCs) and inhibited the proliferation of aNSCs. *Mettl3* depletion not only inhibited neuronal development and skewed the differentiation of aNSCs more toward glial lineage, but also affected the morphological maturation of newborn neurons in the adult brain. m^6^A immunoprecipitation combined with deep sequencing (MeRIP-seq) revealed that m^6^A was predominantly enriched in transcripts related to neurogenesis and neuronal development. Mechanistically, m^6^A was present on the transcripts of histone methyltransferase ***Ezh2***, and its reduction upon *Mettl3* knockdown decreased both Ezh2 protein expression and consequent H3K27me3 levels. The defects of neurogenesis and neuronal development induced by *Mettl3* depletion could be rescued by *Ezh2* overexpression. Collectively, our results uncover a crosstalk between RNA and histone modifications and indicate that Mettl3-mediated m^6^A modification plays an important role in regulating neurogenesis and neuronal development through modulating *Ezh2*.

## Introduction

In the adult mammalian brain, adult neural stem cells (aNSCs) exist in specific regions, namely, the subventricular zone in lateral ventricles and the subgranular zone in the dentate gyrus of the hippocampus [1], [2]. aNSCs can self-renew, and exhibit multipotent capabilities of generating neurons, astrocytes, and oligodendrocytes. The newborn neurons can integrate into existing neural circuits such as those involved in physiological functions including learning and memory [2], [3], [4]. Recent studies have shown that epigenetic modifications, such as DNA modifications, histone modifications, and non-coding RNAs, play essential roles in regulating neurogenesis and neuronal development [5], [6], [7], [8], [9], [10], [11].

*N^6^*-methyladenosine (m^6^A) modification is the most abundant RNA modification in the mRNAs of eukaryotic cells. It is involved in a variety of biological processes including the translation efficiency, degradation, subcellular localization, alternative splicing, and secondary structure of RNA [12], [13], [14], [15], [16], [17], [18], [19], [20], [21]. m^6^A is deposited by methyltransferase-like 3 (Mettl3) and several other components of the methyltransferase complex. It is recognized by its YT521-B homology (YTH) domain-containing proteins and hnRNPA2B1, and is erased by the fat mass and obesity-associated protein (Fto) and the α-ketoglutarate-dependent dioxygenase alkB homolog 5 (Alkbh5). Many studies have revealed that the modulation of m^6^A level is involved in diverse processes including the regulation of fate determination, the proliferation and differentiation of stem cells, homeostasis, DNA damage response, adipogenesis, spermatogenesis, and circadian clock processes [14], [18], [22], [23], [24], [25].

Recently, it has been found that m^6^A is prevalent in mRNAs of the mammalian nervous system, and displays dynamic features during embryonic and postnatal neuronal development [16]. m^6^A eraser *Fto*-deficient mice display impaired neuronal activity and altered behaviors related to dopamine signaling [26], [27], [28]. In addition, the specific knockdown of *Fto* in the mouse medial prefrontal cortex (mPFC) can promote cued fear memory [29]. Our previous study has also found that the constitutive deletion of *Fto* inhibits adult neurogenesis *in vivo*, and impairs spatial learning and memory in mice [30]. Recently, it has been revealed that m^6^A modification regulates axon development [31], [32], and the deletion of *Mettl14* or *Mettl3* dysregulates embryonic cortical neurogenesis [17], [33], postnatal cerebellar development [34], and stress responses in mice [35]. All of these studies suggest important functions of m^6^A modification in the neuronal system. However, the mechanistic role of m^6^A in regulating the proliferation and differentiation of aNSCs remains largely unknown.

In the present study, we found that both *Mettl3* and m^6^A exhibit dynamic and conservative patterns during the differentiation of aNSCs *in vitro*. *Mettl3* depletion significantly reduced m^6^A level and altered the proliferation and cell cycle progression of aNSCs. *Mettl3* depletion also skewed the lineage commitment more toward glia, and inhibited morphological maturation of newborn neurons both *in vitro* and *in vivo*. m^6^A immunoprecipitation combined with deep sequencing (MeRIP-seq) has revealed that m^6^A tags are predominantly enriched in transcripts related to neurogenesis and neuronal development. *Mettl3* depletion specifically dysregulates the expression of genes related to the cell cycle and neuronal development. Finally, we show that *Mettl3* depletion reduces the levels of histone methyltransferase Ezh2 and H3K27me3. The overexpression of *Ezh2* could rescue the defective neurogenesis and neuronal development caused by *Mettl3* depletion. Our results thus uncover a crosstalk between RNA methylation and histone modifications and demonstrate the regulatory role of m^6^A modification in aNSC proliferation and differentiation.

## Results

### *Mettl3* and m^6^A display dynamic expressions during aNSC differentiation

To investigate the role of *Mettl3* in NSC differentiation and neural development, we first isolated aNSCs from the forebrains of adult (2-month-old) wild-type (WT) mice, as described in our previous publications [36], [37]. The cultured aNSCs were positive for the neural stem cell markers *Sox2* and *Nestin* (Figure S1A), and could incorporate thymidine analog 5-bromo-2-deoxyuridine (BrdU) during the proliferation (Figure S1B and C). aNSCs generated β-III tubulin positive (Tuj1^+^) neurons and glial fibrillary acidic protein positive (GFAP^+^) astrocytes upon differentiation (Figure S1D). mRNA levels of multiple pluripotency markers and lineage-specific markers underwent significant alterations during the processes from proliferation to differentiation (Figure S1E). These results indicate the homogeneity, self-renewal capability, and multipotency of the cultured aNSCs.

**Figure 1:**
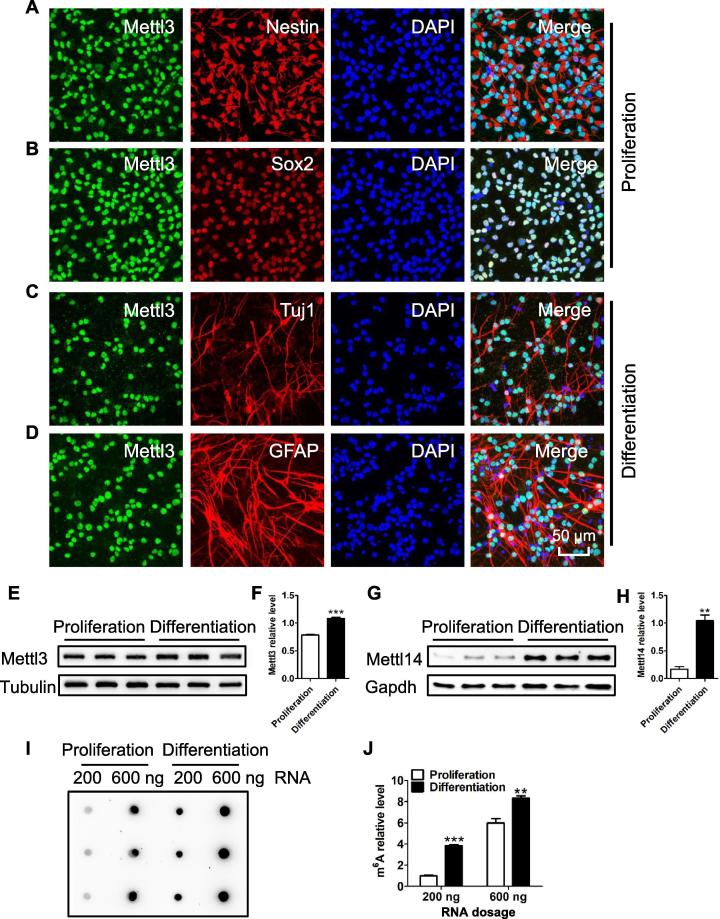
**The dynamic expression of *Mettl3* and m^6^A during the differentiation of aNSCs** Representative immunostaining showing the expression of Mettl3 in Nestin^+^ (**A**) and Sox2^+^ (**B**) aNSCs. Immunofluorescence staining showing the expression of Mettl3 in Tuj1^+^ neuronal cells (**C**) and GFAP^+^ astrocytes (**D**). Western blot (**E**) and quantification (**F**) showing the increased expression of Mettl3 upon the differentiation of aNSCs (*n* = 3). Western blot (**G**) and quantification (**H**) showing the increased expression of Mettl14 upon the differentiation of aNSCs (*n* = 3). Tubulin and Gapdh were used as loading controls. Dot-blot assay (**I**) and quantification (**J**) revealing the increase in m^6^A levels upon the differentiation of aNSCs (*n* = 3). Data are presented as mean ± SEM. Unpaired *t*-test, ^**^, *P* < 0.01; ^***^, *P* < 0.001. Scale bar, 50 μm. aNSC, adult neural stem cell; Tuj1, β-III tubulin; GFAP, glial fibrillary acidic protein.

We then examined the expression of *Mettl3* in aNSCs. We first performed immunofluorescence staining using Mettl3 and Mettl14 specific antibodies, and found that Mettl3 and Mettl14 resided in the nuclei of Nestin^+^/Sox2^+^ aNSCs (Figure 1A and B; Figure S2A and B). Mettl3 and Mettl14 could also be detected in Tuj1^+^ neuronal cells and GFAP^+^ glial cells differentiated from aNSCs (Figure 1C and D; Figure S2C and D). Real-time PCR (RT-PCR) and Western blot results showed a significant increase in the expression of Mettl3 and Mettl14 during aNSC differentiation (Figure 1E–H; Figure S2E). During aNSC differentiation, the expression levels of *Wtap*, which encodes one component of the m^6^A writer complex, and *Fto,* which encodes an m^6^A eraser, were all increased, whereas the expression of *Alkbh5,* which encodes another m^6^A eraser, did not show any significant change (Figure S2F).

Given the dynamic expression of *Mettl3*, we then performed an RNA dot-blot assay to detect m^6^A level. Consistent with the expression pattern of *Mettl3*, we found that the global m^6^A level significantly increased from the proliferation to differentiation stages of aNSCs (Figure 1I and J). Immunostaining also showed that m^6^A existed in the mature neurons (NeuN^+^) of the hippocampus of the mouse brain (Figure S2G), which could be significantly depleted by RNase treatment. These results suggest the specificity of the m^6^A antibody (Figure S2H).

### *Mettl3* regulates the proliferation and differentiation of aNSCs

**Figure 2:**
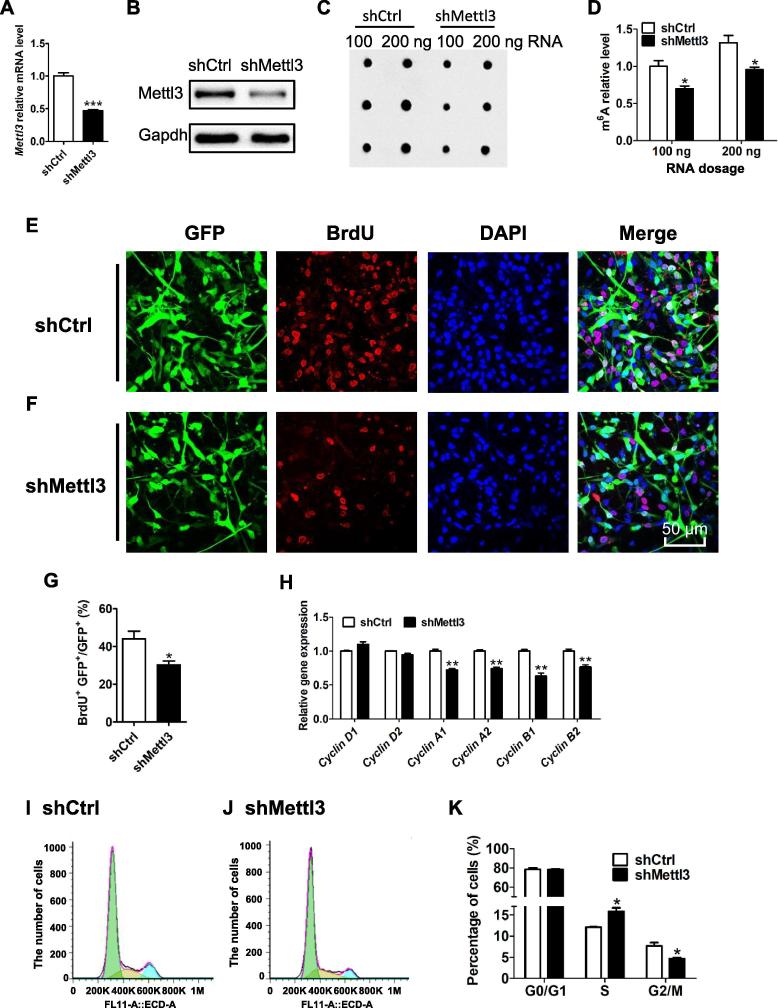
***Mettl3* regulates the proliferation of aNSCs** RT-PCR results showing the mRNA levels of *Mettl3* in control and *Mettl3* KD aNSCs (**A**). *Actin* was used as an internal control (*n* = 3). Western blot assays showing decreased Mettl3 in *Mettl3* KD aNSCs compared to control samples (**B**). Gapdh was used as internal control. Dot-blot assay (**C**) and quantification (**D**) revealing the depletion of m^6^A in *Mettl3* KD aNSCs compared to the control samples (*n* = 3). Representative images of BrdU immunostaining in both control (**E**) and *Mettl3* deficient (**F**) aNSCs. The quantification analysis of BrdU immunostaining in control and *Mettl3* KD aNSCs (*n* = 3) (**G**). The expression levels of multiple cyclin genes in control and *Mettl3* KD aNSCs were detected by qRT-PCR (**H**) (*n* = 3). *Actin* was used as an internal control. Flow cytometry analysis of the cell cycle status of control (**I**) and *Mettl3* KD aNSCs (**J**), and the percentage in each phase (**K**) (*n* = 3). Data are presented as mean ± SEM, unpaired *t*-test, ^*^, *P* < 0.05; ^**^, *P* < 0.01; ^***^, *P* < 0.001. Scale bar, 50 μm. BrdU, 5-bromo-2-deoxyuridine; KD, knockdown.

We next aimed to study the regulatory roles of *Mettl3* in the proliferation and differentiation of aNSCs. We adopted a lentivirus to deliver a short hairpin RNA (shRNA) to knock down *Mettl3* (*Mettl3* KD) in mouse aNSCs (Figure 2A and B). The dot-blot of m^6^A showed that *Mettl3* depletion significantly decreased global m^6^A levels compared to those of the control group (Figure 2C and D). To assess the effect of *Mettl3* deficiency on the proliferation of aNSCs, we first applied a BrdU incorporation assay and found that the number of BrdU^+^ cells was significantly decreased in *Mettl3* KD aNSCs (Figure 2E–G), suggesting the inhibited proliferation of aNSCs. It was noticed that the percentage of Sox2^+^/Nestin^+^ cells and the numbers of cell cycle marker Ki67^+^ cells had not changed upon *Mettl3* KD (Figure S3A–F). Taken together, these data suggest that *Mettl3* deficiency inhibits the proliferation of aNSCs but does not affect their homogeneity.

To further examine the effects of *Mettl3* on the proliferation of aNSCs, we analyzed the expression of multiple cyclins. We found that the mRNA levels of *Cyclin D1* and *D2*, which express throughout the whole cell cycle, did not show any obvious differences between control and *Mettl3* deficient cells (Figure 2H). However, the mRNA levels of *Cyclin A1*, *A2*, *B1*, *B2*, which are specifically expressed in the G2/M phase, were significantly decreased in *Mettl3* deficient aNSCs compared to those of control cells (Figure 2H). Furthermore, we used flow cytometry to analyze the distribution of cells in each phase of the cell cycle. We found that the lack of *Mettl3*increased the number of cells in S phase, but decreased the number of cells in G2/M phase (Figure 2I–K). The quantification of phosphor-histone H3 (p-H3) immunofluorescence staining showed a consistently decreased number of p-H3^+^ cells (Figure S3G). These results indicate that *Mettl3* deficiency dysregulates cell cycle progression.

**Figure 3:**
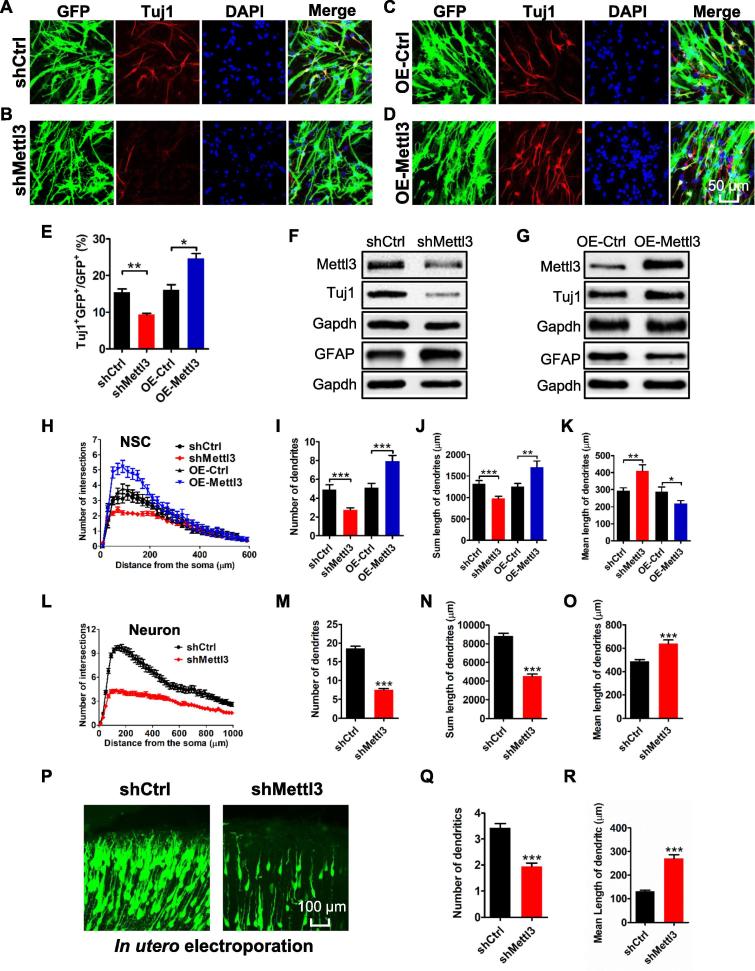
***Mettl3* regulates the differentiation and neuronal development of aNSCs** Representative immunofluorescence images of the differentiated aNSCs with neuronal cell marker Tuj1 of control (**A**, **C**), *Mettl3* KD (**B**), and *Mettl3* OE (**D**) groups. Scale bar, 50 μm. Quantification of Tuj1^+^ cells in differentiated aNSCs with *Mettl3* KD and *Mettl3* overexpression (*n* = 3) (**E**). Western blot assays showing the protein levels of the neuronal cell marker, Tuj1, and astrocyte marker, GFAP, in aNSCs (differentiation condition) with *Mettl3* KD (**F**) and *Mettl3* overexpression (**G**). Sholl analysis of newborn neurons generated upon the differentiation of aNSCs (**H**–**K**) (*n* = 40). Sholl analysis of cultured hippocampal neurons (**L**–**O**) (control group: *n* = 75; KD group: *n* = 66). Representative images of *in utero* electroporation (**P**). The quantifications of the number (**Q**) and length (**R**) of dendrites. Data are presented as mean ± SEM, unpaired *t*-tesst, ^*^, *P* < 0.05; ^**^, *P* < 0.01; ^***^, *P* < 0.001. Scale bar, 50 μm. OE, overexpression.

We next studied the roles of *Mettl3* in regulating the differentiation of aNSCs. Immunofluorescence staining showed that *Mettl3* depletion decreased the number of Tuj1^+^ neurons (Figure 3A and B), but the overexpression of *Mettl3* significantly increased the number of Tuj1^+^ neurons upon the differentiation of aNSCs for 2 days (Figure 3C–E). The results of qRT-PCR and Western blots consistently showed that the depletion of *Mettl3* decreased the level of Tuj1 and increased the level of GFAP (Figure 3F; Figure S4A), and that the overexpression of *Mettl3* led to increased Tuj1 levels and decreased GFAP levels (Figure 3G; Figure S4B).

To further validate the effects of *Mettl3* in regulating aNSC differentiation, we applied a dual luciferase reporter assay to analyze the promoter activity of the neuronal cell marker, *NeuroD1,* and glial cell marker, *Gfap*, in aNSCs. It was found that the overexpression of *Mettl3* could increase the promoter activity of *NeuroD1* and decrease the promoter activity of *Gfap* (Figures S4C and D). To further determine the function of *Mettl3* in neuronal development, we also performed *in utero* electroporation. It was found that the percentage of new born non-neuronal cells (Tuj1^–^GFP^+^/GFP^+^) had significantly increased, meaning the significant decrease in Tuj1^+^GFP^+^ cells, in the cortical plate of *Mettl3* KD mice (Figure S4E and F). These results demonstrate that *Mettl3* regulates lineage commitment during aNSC differentiation, with a preference toward a neuronal fate.

### *Mettl3* regulates neuronal development both *in vitro* and *in vivo*

To assess the effect of *Mettl3* on neuronal development, we further analyzed the morphology of newly born neurons generated upon the differentiation of aNSCs. We observed that both the number of dendritic branches and the total length of dendrites were significantly decreased in new born neurons after *Mettl3* KD (Figure 3H–K; Figure S4G), while they were significantly increased upon *Mettl3* overexpression (Figure 3H–K; Figure S4H). We also performed *Mettl3* KD on cultured hippocampal neurons, and found that *Mettl3* deficient neurons showed a decreased number of intersections and dendrites, a reduced total length of dendrites, and an increased mean length of dendrites (Figure 3L–O; Figure S4I). Knocking down of *Mettl3* by *in utero* electroporation consistently resulted in the number of dendrites per neuron also being significantly decreased and the mean length of dendrites being increased (Figure 3P–R). These results suggest that *Mettl3* promotes neuronal development both *in vitro* and *in vivo*.

### m^6^A modified transcripts are involved in neurogenesis and neuronal development

**Figure 4:**
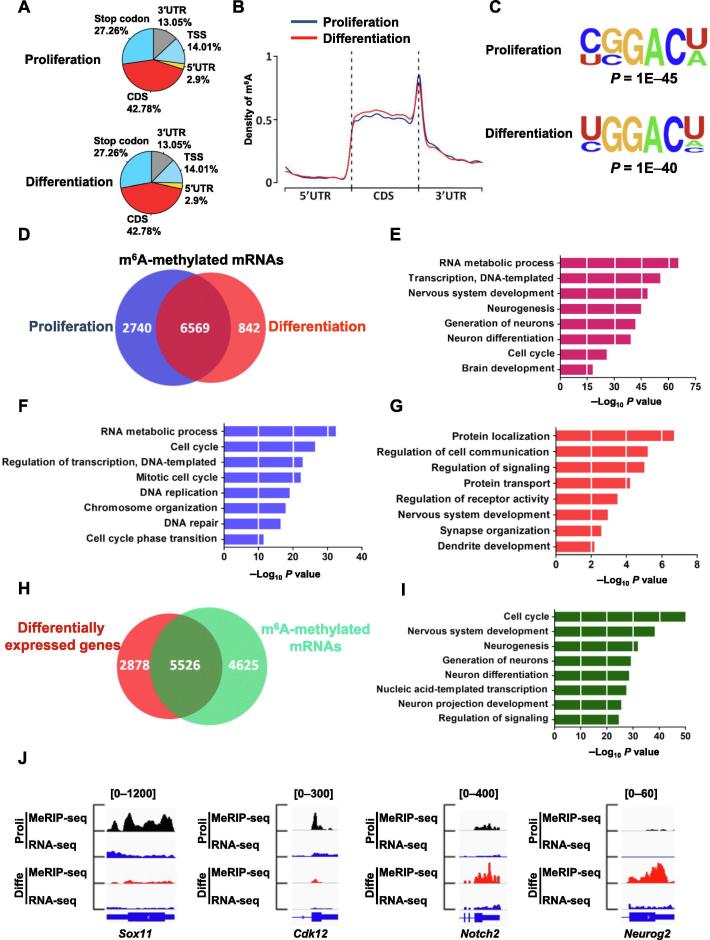
**Dynamic m^6^A modification from proliferation to differentiation of aNSCs** Transcriptome-wide distribution of m^6^A peaks in aNSCs under the conditions of proliferation and differentiation (**A**, **B**) (*n* = 2). The most common sequence motif among m^6^A peaks during proliferation and differentiation (**C**). Venn diagram illustrating the m^6^A modified genes in proliferating and differentiated samples (**D**). GO analysis of the common m^6^A modified genes shared between proliferating and differentiated samples (**E**). GO analysis of the m^6^A modified specific genes identified in proliferating samples (**F**) and differentiated samples (**G**). Venn diagram illustrating the total m^6^A-tagged genes identified in proliferating and differentiated samples, and differentially expressed genes identified in differentiated samples compared to proliferating samples (**H**). GO analysis of the common genes shared between m^6^A tagged genes and differentially expressed genes (**I**). IGV tracks showing several genes with differential m^6^A modification from the proliferation to differentiation of aNSCs (**J**). RNA-seq reads were used as input. TSS, transcription start site; GO, Gene Ontology; Proli, proliferation; Diffe, differentiation; IGV, Integrative Genomics Viewer.

To systematically illuminate the function of m^6^A in neurogenesis and neuronal development, we performed m^6^A immunoprecipitation combined with deep sequencing (MeRIP-seq) to detect the m^6^A peaks and explore their distribution in the transcriptome of aNSCs under proliferation and differentiation conditions (Figure S5A). We observed in both proliferating and differentiated aNSC samples that the m^6^A peaks were predominantly located in the coding sequence (CDS) and that the 3′ untranslated regions (3′UTR) were especially enriched near stop codon regions (Figure 4A and B). The m^6^A distribution pattern displayed a high degree of similarity between proliferation and differentiation conditions, and was enriched in the m^6^A consensus motif, which was consistent with previous reports [15], [18] (Figure 4C). Bioinformatic analysis revealed that 9309 and 7411 m^6^A methylated mRNAs were detected in proliferation and differentiation conditions, respectively (Table S1), whereas 6569 m^6^A methylated mRNAs overlapped between the two conditions (Figure 4D). Gene ontology (GO) analysis showed the functional enrichment of these 6569 overlapped genes was related to the transcription, neurogenesis, neuronal differentiation and cell cycle related pathways (Figure 4E). Furthermore, 2740 unique m^6^A methylated RNAs in proliferating samples were enriched in terms related to DNA replication and the cell cycle, while 842 unique m^6^A methylated genes in differentiated samples were enriched in terms related to neurogenesis, neuronal development and differentiation (Figure 4F and G).

To determine the relationship between m^6^A modification and gene expression, we next performed RNA-seq in these two conditions to uncover any global transcriptome alterations (Figure S5B). It was found that 8404 genes displayed altered expression from proliferation to differentiation conditions of aNSCs: 4378 (52.09%) mRNAs were up-regulated while 4026 (47.91%) genes were down-regulated (Table S2). Among these 8404 genes, 5526 (65.75%) genes were modified by m^6^A, including 3065 (55.47%) up-regulated genes and 2461 (44.53%) down-regulated genes (Figure 4H; Table S3). GO analysis showed that these 5526 genes were enriched in pathways related to the cell cycle, nervous system development and neuronal differentiation (Figure 4I). Key examples include, *Sox11* and *Cdk12*, involved in proliferation, and *Notch2* and *Neurog2*, related to neuronal differentiation (Figure 4J; Figure S5C). Taken together, these data suggest that m^6^A modification plays a key role in regulating the gene expressions of aNSCs.

### *Mettl3* regulates m^6^A modification of neurogenesis-related genes

**Figure 5:**
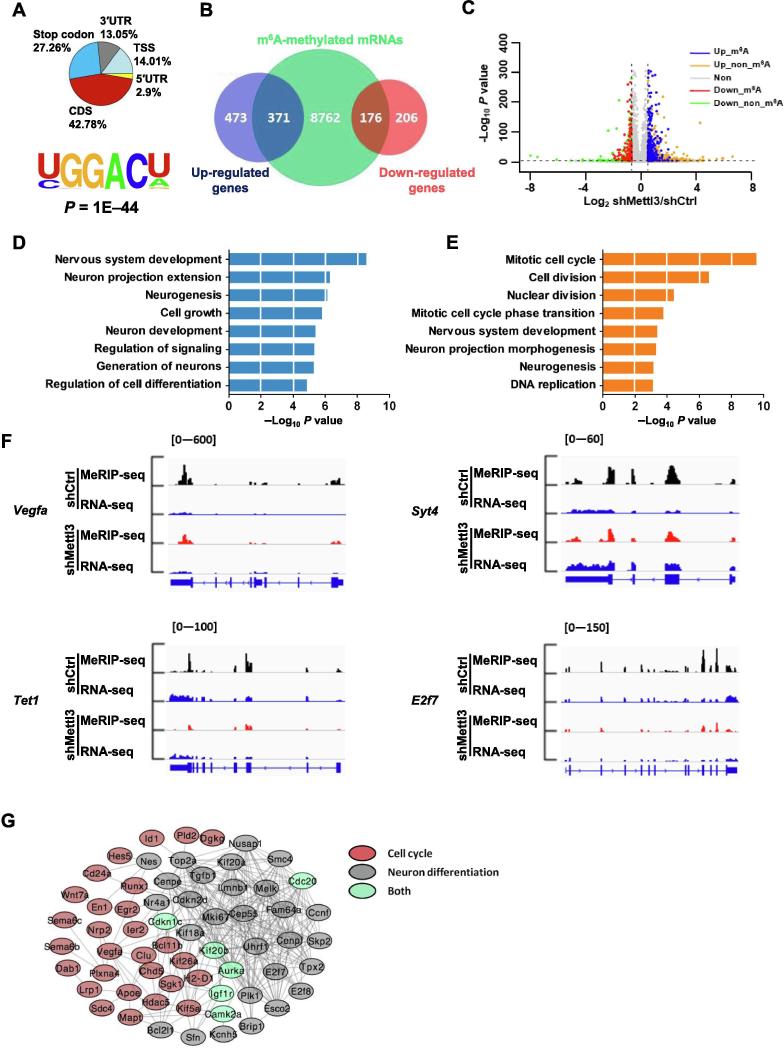
**Mettl3-mediated m^6^A regulates gene expression in proliferating aNSCs** Transcriptome-wide distribution of m^6^A peaks in *Mettl3* KD samples (*n* = 2) (**A**). Venn diagram illustrating the up-regulated and down-regulated genes and m^6^A-modified transcripts in *Mettl3* KD samples (**B**). Volcano plot showing differentially expressed genes between control and *Mettl3* KD samples (**C**). GO analysis of m^6^A-tagged up-regulated (**D**) and down-regulated (**E**) transcripts in *Mettl3* KD samples. Four examples of IGV tracks showing that the enrichment of m^6^A in several transcripts, which are related to neurogenesis and neuronal development, were significantly decreased in *Mettl3* KD samples (**F**). RNA-seq reads were used as input. The interaction network showing differentially expressed transcripts with m^6^A modification. Genes related to cell cycle and neuronal differentiation are marked in red and gray, respectively, whereas genes related to both processes are marked in green (**G**).

Given that *Mettl3* regulates neurogenesis and neuronal development, we next sought to investigate whether *Mettl3* KD affected gene expression related to those two areas. We first examined m^6^A distribution patterns in control and *Mettl3* KD aNSCs (in a proliferation condition) (Figure S5A), and observed that *Mettl3* KD had not altered the overall m^6^A distribution patterns in transcripts compared to those of the control samples (Figure 5A). Furthermore, m^6^A was enriched in the same m^6^A consensus motifs of both *Mettl3* KD and control samples, even though the global m^6^A level had been significantly decreased (Figure 2C; Figure 4C; Figure 5A).

To examine the effects of *Mettl3* depletion on gene expression, we performed RNA-seq in control and *Mettl3* KD samples (a proliferating condition) (Figure S5B). RNA-seq data analysis showed that a total of 1226 genes exhibited altered expressions in *Mettl3* KD samples compared with those of control samples (Fold change >1.5), including 844 (68.84%) up- and 382 (31.16%) down-regulated genes (Figure 5B; Table S4). Among the up-regulated genes, 371 genes (43.96%) were methylated by m^6^A, while 176 down-regulated genes (46.07%) were methylated by m^6^A (Figure 5B and C). GO analysis showed that the m^6^A tagged up-regulated genes were enriched in areas of neuronal differentiation, neurogenesis and nervous system development (Figure 5D and E). The representative IGV images showed that m^6^A tagged down-regulated genes were enriched in terms related to the neuronal development, such as *Vegfa* and *Syt4,* and cell cycle, cell proliferation, such as *Tet1* and *E2f7* (Figure 5F). The analysis using STRING database showed the interaction between proteins coded by m^6^A tagged transcripts (Figure 5G). Meanwhile, we also performed GO analysis of differentially expressed genes without m^6^A modification, and found that these genes showed low correlations with neuronal development and the cell cycle (Figure S5E and F). Together, these results suggest that altered m^6^A modification induced by *Mettl3* depletion impacts the expression of genes related to cell cycle progression and neuronal development.

### *Ezh2* rescues the deficits of neuronal development and neurogenesis induced by *Mettl3* depletion

**Figure 6:**
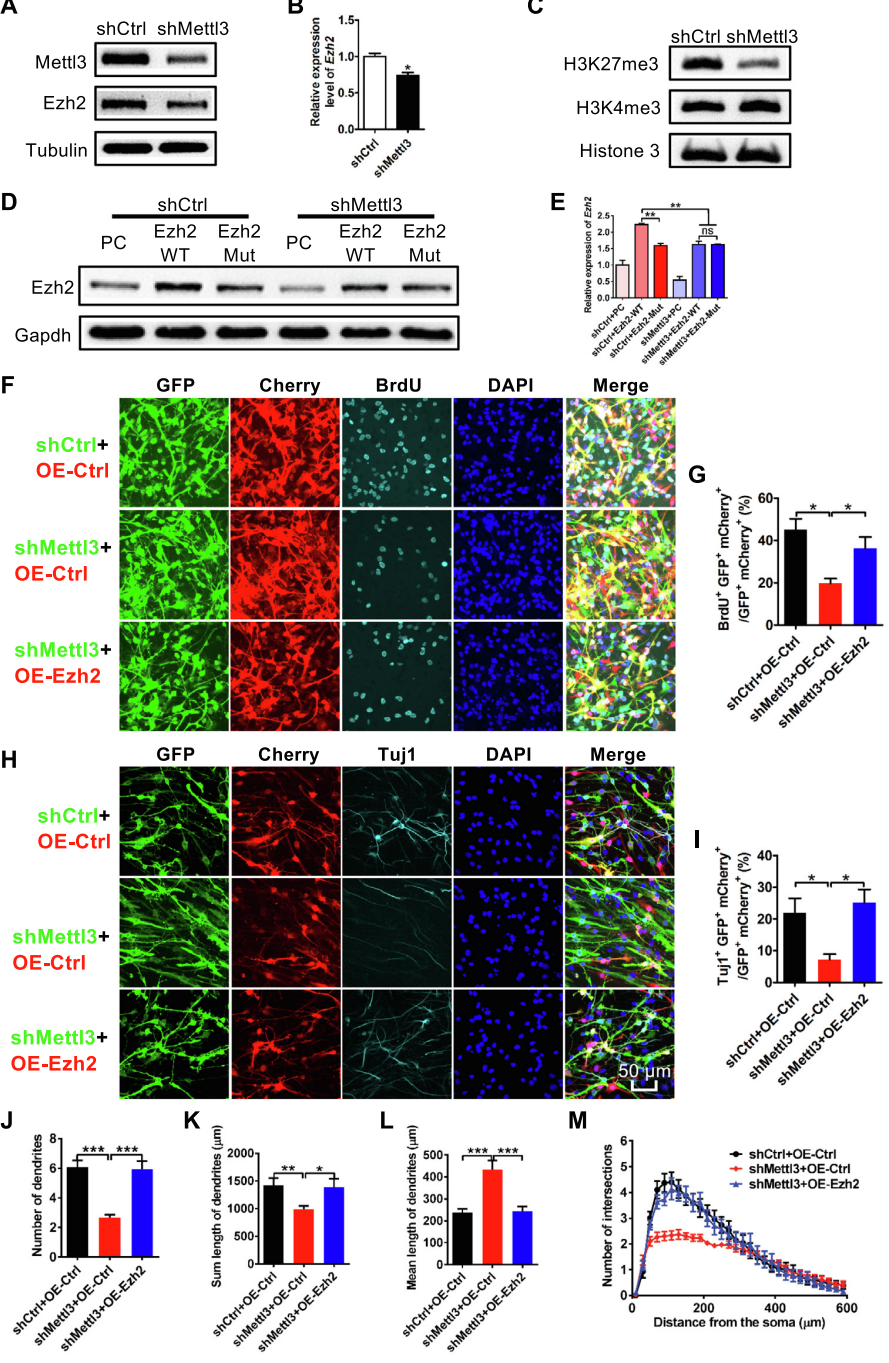
**Ezh2 is regulated by Mettl3 and can rescue the deficits of neuronal development and neurogenesis induced by *Mettl3* knockdown** Western blot assay showing *Mettl3* KD led to a significant decrease in Ezh2 (**A**, **B**), and H3K27me3 (**C**) at the protein level (*n* = 3). Western blot assay (**D**) and quantification (**E**) showing that *Mettl3* KD significantly decreased expression of WT Ezh2, but had less effect on the expression of mutant Ezh2 (*n* = 3). Representative images (**F**) and quantification (**G**) showing that the overexpression of *Ezh2* rescued the reduced proliferation induced by *Mettl3* KD (*n* = 3). Representative images (**H**) and quantification (**I**) showing that the overexpression of *Ezh2* rescued the inhibited neuronal differentiation induced by *Mettl3* KD (*n* = 3). *Ezh2* rescued the impaired morphological maturation of newborn neurons induced by *Mettl3* KD (*n* = 30) (**J**–**M**). Data are presented as mean ± SEM, unpaired *t-*test, ^*^, *P* < 0.05; ^**^, *P* < 0.01; ^***^, *P* < 0.001. Scale bar, 50 μm.

One recent study has shown that m^6^A regulates specific histone modifications [38]. Through analyzing m^6^A sequencing data, we observed that the transcripts of histone methyltransferase *Ezh2*, which plays important roles in neurogenesis and neuronal development [37], [39], [40], were tagged with m^6^A modification (Figure S6A). The region with the largest m^6^A peak was on exon 10 of *Ezh2* and this was validated by m^6^A RIP followed by qPCR (Figure S6B). m^6^A enrichment at *Ezh2* was significantly decreased upon *Mettl3* depletion, as confirmed by m^6^A-IP-qPCR (Figure S6C). Western blot results showed that the protein level of *Ezh2* was significantly decreased upon *Mettl3* depletion, whereas its mRNA level did not change (Figure 6A and B; Figure S6D). Consistently, the protein level of H3K27me3 was also decreased after *Mettl3* depletion while no observable changes were noted in the levels of H3K4me3 (Figure 6C). We further constructed a mutant *Ezh2* plasmid (the site within the biggest m^6^A peak on exon 10 was denoted in Figure S6A and was mutated). The overexpression of WT or mutant *Ezh2* showed similar transcription efficiencies (Figure S6E and G), and neither of them were observed to affect any protein and mRNA levels of Mettl3 in either aNSCs or N2a cells (Figure S6F; Figure S6H and I). Consistent with the effects of *Mettl3* KD on the expression of *Ezh2*, *Mettl3* overexpression not only significantly increased the global level of m^6^A, but also upregulated the levels of Ezh2 and H3K27me3 (Figure S6J–L). These results suggest that *Mettl3* regulates *Ezh2*, but not *vice versa*.

Next, we analyzed the effect of knocking down *Mettl3* on the expression of exogenous *Ezh2*. We found that the protein levels of both endogenous and exogenous WT Ezh2 were significantly decreased upon *Mettl3* KD (Figure 6D and E), while mRNA levels remained unaltered (Figure S6D). However, *Mettl3* knockdown exhibited less effect on the protein level of mutated *Ezh2* (Figure 6D and E). Together, these results suggested *Mettl3* deposited m^6^A modification regulates Ezh2 expression at the translational level.

We finally examined whether *Ezh2* could rescue the deficits of aNSCs induced by *Mettl*3 depletion. The results showed that the overexpression of *Ezh2* could increase proliferation (indicated by the number of BrdU^+^ cells) (Figure 6F and G) and promote neuronal differentiation (indicated by the number of Tuj1^+^ cells) of aNSCs induced by *Mettl3* KD (Figure 6H and I). Furthermore, the dendritic number and length, and the number of intersections of new born neurons, were also increased upon *Ezh2* overexpression (Figure 6J–M). Taken together, these results indicate that overexpression of *Ezh2* could rescue the deficits of neurogenesis and neuronal development induced by *Mettl3* depletion.

## Discussion

Adult neurogenesis is a multi-step event involving the maintenance of the stem cell pool, lineage commitment, maturation, and the establishment of neural circuits. All of these processes are precisely and intensively regulated by genetic and epigenetic mechanisms. Here, we have demonstrated the important roles of Mettl3-mediated m^6^A methylation in regulating the neurogenesis and neuronal development through modulating the expression of histone methyltransferase Ezh2 (Figure S6M).

m^6^A has been shown to modulate the self-renewal, differentiation, and lineage determination of multiple stem cell types by regulating gene expression, especially the expression of key transcription regulators [18], [23], [25], [41], [42]. The distribution pattern of m^6^A is highly conservative with an abundance in 3′UTRs and near the stop codons observed across different cell types [16], [18], [19]. Our results have shown that, in the transcriptomes of aNSCs, m^6^A exhibits similar distribution features, and is enriched at similar motifs. These highly conservative features of m^6^A modification indicate its important function in different cell types.

Dynamic m^6^A modification has not only been found during neuronal development, but also can be induced by neuronal activity and learning, pointing to the function of m^6^A in the neuronal system [16], [26], [27], [29], [43]. Our previous study showed that the deletion of *Fto* inhibits adult neurogenesis as well as learning and memory through its regulation of *BDNF* [30]. The deletion of either *Mettl3*, *Mettl14*, or *Ythdf2* could disturb embryonic cortical neurogenesis in mice [17], [33]. Ectopic expression of *Mettl3* induced neuronal defects in the cerebellum [34]. Our present results indicate that *Mettl3* depletion not only inhibits the proliferation and cell cycle progression of aNSCs, also skews their lineage commitment more toward glia during the differentiation *in vitro*. Moreover, our study also showed that m^6^A modification regulates the morphological development of neurons. We found that, besides the RNAs tagged by m^6^A that are highly overlapping between proliferation and differentiation conditions, m^6^A also specially tags unique mRNAs under either proliferation or differentiation conditions. It is plausible to speculate that the dynamic and specific distribution patterns of m^6^A tagging on transcripts are highly correlated to its function in aNSCs. In this way, these results further highlight the function of m^6^A modification in neuronal development and adult neurogenesis.

The roles exactly played by m^6^A modification are under intensive study. Previous studies have reported that m^6^A regulates gene expression through modulating splicing and the efficiency of translation [19], [44], [45], [46]. Although Mettl14 itself does not have catalytic activity, the deletion *of Mettl14* could significantly reduce global m^6^A levels, disturb embryonic cortical neurogenesis, impair striatal-mediated behavior and function through affecting global transcriptomes [17], [47]. All these studies indicate the roles of RNA methyltransferase-mediated m^6^A in regulating gene expression during development and during the operation of various physiological functions such as that of neuronal function. One recent study has indicated that m^6^A modification affects histone modification. *Mettl14* haploinsufficiency led to genome-wide changes in histone modification including increased H3K27me3, H3K27ac, and H3K4me3 [38]. However, we observed that the level of the methyltransferase Ezh2 was significantly decreased in *Mettl3* deficient cells, while its overexpression could rescue *Mettl3* deficiency induced phenotypes. Whether this discrepancy was due to the specific cell types under study requires further examination.

In summary, our study has revealed the critical roles of *Mettl3*-mediated m^6^A modification in regulating cell cycle progression, lineage commitment and neuronal development of aNSCs through modulating histone methyltransferase Ezh2. These findings highlight the function of epitranscriptomic mechanisms in neuronal development and adult neurogenesis.

## Materials and methods

### Animals

Mice were housed in standard conditions in the Laboratory Animal Center of Zhejiang University on a 12 h light/dark schedule with free access to food and water. The pregnant mice were purchased from Shanghai SLAC Laboratory Animal Company, China. All animal experiments were conducted according to protocols approved by the Zhejiang University Animal Care and Use Committee.

### Isolation and culture of aNSCs

The isolation of NSCs from the forebrain of adult mice was performed as described previously [36], [37]. aNSCs were cultured in DMEM/F-12 medium containing 20 ng/ml FGF-2 (Catalog No. 100-18B-B; PeproTech, Rocky Hill, NJ), 20 ng/ml epidermal growth factor (Catalog No. 100-15; PeproTech), 2% B27 supplement (Catalog No. 12587-010; Thermo Fisher Scientific, Grand Island, NY), 1% antibiotic–antimycotic (Catalog No. 15140-122; Thermo Fisher Scientific), and 2 mM l-glutamine (Catalog No. 25030–149; Thermo Fisher Scientific) in a 5% CO_2_ incubator at 37 °C.

### *In vitro* proliferation and differentiation assay

For the *in vitro* proliferation assay, aNSCs were cultured on coverslips with medium supplied with 5 µM BrdU for 8 h. For the *in vitro* differentiation assay, aNSCs were cultured on coverslips with proliferation medium, and then transferred into differentiation medium containing 1 µM retinoic acid (Catalog No. R-2625; Sigma, Saint Louis, MO) and 5 µM forskolin (Catalog No. F-6886; Sigma) for 48 h.

### Immunofluorescence staining

After washing with PBS for 30 min, cell samples on coverslips or brain sections were blocked with 3% normal goat serum and 0.1% triton X-100 in PBS for 1 h at room temperature. Samples were incubated with primary antibodies over night at 4 °C. The second day, cells or sections were washed with PBS for 30 min, and then incubated with Fluorophore-conjugated secondary antibodies for 1 h at room temperature. For BrdU immunostaining, samples were pretreated with 1 M HCl at 37 °C for 30 min before the application of block solution. The following primary antibodies were used: Mettl3 (Catalog No. 21207-1-AP; Proteintech, Rosemont, IL), Mettl14 (Catalog No. HPA038002; ATLAS, Bromma, Sweden), m^6^A (Catalog No. 202003; Synaptic Systems, Goettingen, Germany), NeuN (Catalog No. AB2237; Millipore, Burlington, MA), Nestin (Catalog No. 556309; BD Pharmingen, San Jose, CA), SOX2 (Catalog No. sc-365823X; Santa Cruz Biotechnology, Dallas, TX, USA), Tuj1 (Catalog No. G712A; Promega, Madison, WI), GFAP (Catalog No. Z0334; DAKO, Santa Clara, CA), BrdU (Catalog No. ab6326; Abcam, Cambridge, MA), and Ki67 (Catalog No. AB9260; Millipore).

### Electroporation and luciferase assays

Electroporation was performed with an electroporator (Catalog No. AAB-1001; Amaxa Lonza, Germany) as described previously [37]. Briefly, the cultured aNSCs were collected and resuspended with 100 µl nucleofection solution and electroporated using the manufacturer’s protocol. The electroporated cells were cultured with fresh proliferation medium and the medium was replaced with differentiation medium (5 µM forskolin and 1 µM retinoic acid) on the second day. 48–60 h later, the cells were collected for a luciferase assay with a luminometer according to the manufacturer’s protocol (Promega). 0.1 µg Renilla-luciferase plasmids and 2 µg NeuroD1-/Gfap-luciferase plasmids were used for each electroporation, respectively. To knock down *Mettl3*, short hairpin RNA (shRNA) targeting *Mettl3* (5′-taagcacactgatgaatcttt-3′, Qiagen, Hilden, Germany) was cloned into to lentivirus-U6 vectors.

### Total RNA isolation, reverse transcription, and quantitative real-time PCR

Total RNA was extracted from aNSCs using TRIzol reagent (Catalog No. 15596018; Thermo Fisher Scientific). Total RNA was isolated and the concentration was quantified using a NanoDrop spectrophotometer 2000 (Thermo Fisher Scientific). 0.5 µg total RNA was used for reverse transcription (RT) using a RT reagent kit (Catalog No. R223-01; Vazyme, Nanjing, China). Quantitative real-time PCR (qRT-PCR) was performed using SYBR Green (Catalog No. Q71502; Vazyme). All real-time PCR reactions were performed in triplicate, and the results were analyzed using the ^ΔΔ^Ct method.

### Western blot

The collected cells were washed with PBS and resuspended with RIPA buffer (Catalog No. ab156034; Abcam, Cambridge, MA, USA) containing 1× protease inhibitor cocktail (Catalog No. 04693124001; Sigma, Saint Louis, MO, USA). The samples were centrifuged at 4 °C for 20 min at 14,000 rpm and the supernatants were collected. Samples were then denatured for 5 min at 95 °C and then subjected to SDS–polyacrylamide gel electrophoresis. The following primary antibodies were used: anti-Mettl3 (Catalog No. 21207-1-AP; Proteintech), anti-Mettl14 (Catalog No. HPA038002; ATLAS), anti-Tuj1 (Catalog No. G712A; Promega), anti-GFAP (Catalog No. 3670; Cell Signaling, Danvers, MA), anti-tubulin (Catalog No. ab15246; Abcam), anti-Ezh2 (Catalog No. 3147; Cell Signaling), anti-H3K27me3 (Catalog No. 07-449; Millipore), anti-H3K4me3 (Catalog No. ab8580; Abcam), anti-Histone3 (Catalog No. ab1791; Abcam), and anti-Gapdh (Catalog No. AM4300; Thermo Fisher Scientific). Secondary HRP conjugated antibodies were applied for 1 h at room temperature. The signal was detected by Tanon Detection system (Tanon 5200, Shanghai, China) and the intensity of immuno-blot bands was normalized to those of Gapdh or Tubulin.

### m^6^A dot-blot assay

For m^6^A dot-blot, total RNA samples were denatured at 65 °C, and then spotted onto Hybond N^+^ membranes (Catalog No. NP1096; GE Healthcare, Buckinghamshire, UK). Membranes were blocked with 5% milk for 1 h at room temperature, and then incubated with primary antibodies overnight at 4 °C. On the next day, membranes were incubated with HRP-conjugated secondary antibodies for 30 min at room temperature. The signal was detected by Tanon Detection system, and the signal density was quantified using Photoshop software.

### Cell cycle analysis

To analyze the cell cycle aNSCs, propidium iodide (PI) staining was performed according to the manufacturer’s instructions (Multi Sciences, Hangzhou, China). In brief, after subculture for 24 h, aNSCs were harvested and fixed with absolute ethanol. The pellet was then dislodged in PBS and stained with propidium iodide solution at room temperature for 30 min. The cells were then analyzed using CytoFLEX (Beckman Coulter, Boulevard Brea, CA) and data were analyzed using FlowJo software.

### *In utero* electroporation (IUE)

*In utero* electroporation was performed as described previously [48]. Briefly, the timed pregnant C57 mice (E13.5) were anesthetized using isoflurane. The uterine horns were then exposed and bathed in warm PBS. 2 μl recombinant plasmid (final concentration 1.5 mg/ml) mixed with fast green (0.01%) was manually microinjected into the lateral ventricle with a glass micropipette (Hirschmann DE-M 16). For electroporation, five 100-microsecond pulses of 35 V with a 900-microsecond interval were delivered across the uterus using an electroporator (Catalog No. 101438; BEX, Tokyo, Japan). After electroporation, the uterine horns were placed back into the abdominal cavity to allow the embryos to continue normal development. The pregnant mice were sacrificed at scheduled time points as indicated and the embryos were harvested for further analysis.

### mRNA purification, m^6^A MeRIP-seq and m^6^A MeRIP-qPCR

m^6^A MeRIP-seq was carried out as previously described with some modifications [16], [25], [49]. In brief, mRNAs were purified from total RNAs using a Dynabeads® mRNA purification kit (Catalog No. 61006; Thermo Fisher Scientific), and digested with DNase I to remove any potential DNA contamination. mRNAs were fragmented to around 100 nt using an RNA fragmentation reagent (Catalog No. AM8740; Thermo Fisher Scientific) through incubation at 90 °C for 1 min, and were then precipitated with ethanol. The m^6^A polyclonal antibody (Catalog No. 202003; Synaptic Systems) was incubated with 40 μl Dynabeads™ Protein A (Catalog No. 10001D; Thermo Fisher Scientific) in 500 μl IPP buffer (150 mM NaCl, 0.1% NP-40, 10 mM Tris-HCl, pH 7.4) for 1 h at room temperature. The recovered mRNAs were denatured at 75 °C for 5 min and put on ice immediately. 5 μg of fragmented mRNAs were added to the antibody-bead mixture followed by the incubation at 4 °C for 4 h. After extensive washing by IPP buffer five times, the m^6^A-containing RNAs were eluted for twice with 300 μl 0.5 mg/ml *N*^6^-methyladenosine (Catalog No. P3732; Berry & Associates, Dexter, MI) at room temperature for 1 h using gentle rotation. The eluted samples were combined together and extracted with Acid Phenol (pH 4.3–4.7), followed by standard ethanol precipitation. The recovered RNA was subjected to the cDNA library construction by using KAPA Stranded mRNA-Seq Kit Illumina® platform (Catalog No. KK8401; KAPA, Boston, MA) and sequenced on the HiSeq 3000 platform.

### RNA-seq and MeRIP-seq data analysis

MeRIP-seq and RNA-seq was performed. The proliferation (proli) versus differentiation (differ) cells, and control versus *mettl3* knockdown cells were conducted in two biological replicates using an Illumina HiSeq 3000 platform. Raw reads of each sample (*n* = 2 for each group) were trimmed using the Trimmomatic software for each sample to remove adaptor sequences and bases with low quality [50]. The processed reads with length larger than 35 nt were then aligned to the mouse reference genome (version mm10, UCSC) using TopHat2 [51] with default parameters. Only unique mapped reads with mapping quality no less than 20 were kept for the subsequent analysis.

For MeRIP-seq, the MACS2 software was used to identity m^6^A-enriched (version 2.0.10) [52], with the corresponding input sample serving as control. MACS2 was run with default options except for ‘-nomodel, -keepdup all’ to turn off fragment size estimation and to keep all uniquelymapping reads, respectively. To identify high confidential or overlapped m^6^A peaks, peaks were intersected in a pairwise fashion among two replicates or between two conditions using the BedTools package by setting ‘-f 0.5’ [53]. Fold changes for m^6^A peaks were obtained from MACS2 output.

For RNA-seq, the number of reads mapped to each Ensemble gene (release 68) were counted using the HTSeq python package [54], with the ‘union’ overlap resolution mode and unstranded count feature by setting ‘--mode = union’ and ‘--stranded = no’, respectively. The expression of transcripts was quantified as reads per kilobase of exon model per million mapped reads (RPKM). These RNAs, which are methylated by m^6^A in both conditions, are defined as overlapped m^6^A methylated RNAs.

### Motif identification within m^6^A peaks

Motifs enriched in m^6^A peaks within all mRNAs were identified using HOMER software (v4.7) [55]. BEDTools' shuffleBed (version 2.16.2) was used to generate random peaks within total mRNAs as background sequences [53]. The motif length was restricted to 5–6 nucleotides.

### Statistical analysis of differentially expressed genes

To identify differentially expressed genes, the R-package DEGseq was used with fold-change ≥1.5, *P* ≤1 × 10^−3^ and the method MARS (MA-plot-based method with random sampling model) as the parameters [56].

### Gene ontology analysis

Gene ontology (GO) analysis was performed using the DAVID database [57]. Enrichment maps were generated by Cytoscape (version 3.5.0) with the Enrichment Map plugin [58]. Each enriched GO function term is represented by a node and the node size is proportional to the number of genes in its corresponding function term in the enrichment maps. The thickness of each edge represents the number of common genes between two linked nodes. Similar GO functions are categorized as one cluster. The function term and the number of genes in each cluster are labeled. A gene interaction network was generated using STRING [59].

### Statistical analysis

All data are expressed as mean ± SEM. GraphPad Prism (GraphPad Software Inc.) was used for statistical analysis. Unpaired student’s *t*-test was used to determine the differences between two groups; a two-way ANOVA analysis followed by Bonferroni multiple-comparison test was used to determine differences between multiple groups. *P* < 0.05 was considered statistically significant.

## Data availability

The raw sequence data reported in this paper have been deposited in the Genome Sequence Archive [60] in BIG Data Center [61], Beijing Institute of Genomics (BIG), Chinese Academy of Sciences, as GSA: CRA001248, which is publicly accessible at http://bigd.big.ac.cn/gsa.

## Authors’ contributions

XL and QS conceived and designed the project. JC, HS, XC, and Y-JZ performed aNSC culture, neurogenesis assay, m^6^A dot-blot, RT-PCR, and Western blot assays. JC, YZ, CH, and BS performed RNA-seq and MeRIP-seq data analysis. YY did RNA-seq and MeRIP-seq library construction. XL, JC, YZ, BS, YY, YGY, and QS analyzed the data. XL and YY wrote the manuscript with input from all other authors. All authors had read and approved the final manuscript.

## Competing interests

The authors declared no competing financial interests.

## Supplementary material

Supplementary Figure S1 **Validation of the homogeneity, self-renewal capability and multipotency of aNSCs** Representative immunofluorescence staining showing that the cultured aNSCs were positive for NSC markers Nestin and Sox2 (**A**). BrdU staining revealed the self-renewal capability of Nestin^+^ (**B**) and Sox2^+^ (**C**) aNSCs. Upon differentiation, aNSCs generated neuronal cells (Tuj1^+^) and astrocytes (GFAP^+^) (**D**). qRT-PCR analysis of mRNA levels of multiple cell markers *Nestin* (NSC marker), Tuj1 and *NeuroD* (neuronal markers), *Gfap* and *S100β* (glial markers) in proliferation and differentiation conditions of aNSCs (**E**) (*n* = 3). *Actin* mRNA was used as an internal control. Data are presented as mean ± S.E.M., unpaired *t*-test, ^*^*P* < 0.05; ^**^*P* < 0.01; ^***^*P* < 0.001. Scale bar, 50 μm.

Supplementary Figure S2 **Expression of m^6^A modification-related multiple genes in cultured aNSCs and detection of m^6^A in brain** Representative immunofluorescence staining of Mettl14 with aNSC markers Sox2 (**A**), Nestin (**B**), and the neuronal cell marker Tuj1 (**C**) and astrocyte marker Gfap (**D**). qRT-PCR analysis of *Mettl3* and Mettl14 mRNA levels between the proliferation and differentiation of aNSCs, respectively (**E**). The comparison of mRNA levels of *Wtap*, *Fto* and *Alkbh5* between the proliferation and differentiation of aNSCs (**F**). Representative immunofluorescence staining showing that m^6^A was enriched in mature neurons (NeuN^+^) in the hippocampus of postnatal day 14 (P14) mouse brains (**G**). RNase treatment significantly depleted the signal intensity of m^6^A immunofluorescence staining (**H**). Scale bar through A–D and G, H, 50 μm.

Supplementary Figure S3 **Effects of *Mettl3* knockdown on the homogeneity and proliferation of aNSCs** Representative immunofluorescence staining of NSC marker Sox2 in control and Mettl3 KD aNSCs (**A**). Representative immunofluorescence staining of NSC marker Nestin in control and *Mettl3* KD aNSCs (**B**). Representative immunofluorescence staining of cell cycle marker Ki67 in control and *Mettl3* KD aNSCs (**C**). Quantification results showed that Mettl3 KD did not affect the percentage of Sox2^+^ (**D**), Nestin^+^ (**E**) and Ki67^+^ (**F**) cells compared to control groups, respectively (*n* = 3). Quantitative analysis showed *Mettl3* KD induced the decrease of phosphor-histone H3 positive (p-H3^+^) cells (**G**) (*n* = 3). Data are presented as mean ± S.E.M., unpaired *t*-test, ^*^*P* < 0.05; ^**^*P* < 0.01; ^***^*P* < 0.001. Scale bar, 50 μm.

Supplementary Figure S4 **Effects of Mettl3 knockdown and overexpression on the differentiation of aNSCs and neuronal development** qRT-PCR results showed the mRNA levels of *Tuj1*, *NeuroD*, *Gfap* and *S100β* in the control and *Mettl3* KD samples (differentiation conditions) (**A**) (*n* = 3). qRT-PCR results showed the mRNA levels of *Mettl3*, *Tuj1*, *NeuroD*, *Gfap* and *S100β* in the control and *Mettl3* over-expression samples (differentiation conditions) (**B**) (*n* = 3). Luciferase assay results show that the overexpression of *Mettl3* had increased the promoter activities of the pan-neuronal marker, *NeuroD* (**C**), but decreased the promoter activities of glial cell marker, Gfap (**D**) (*n* = 3). Representative immunofluorescence images of *in utero* electroporation (**E**). The control or *Mettl3*-shRNA plasmid was electroporated into E13.5 embryonic mouse brains, sacrificed at E14.5, and the embryonic brains were sectioned with Cryostat. The harvested sections underwent immunofluorescence staining with images taken using an Olympus confocal microscope (at least five sections from each brain, 2 pups from each litter and 2 litters were used each group). Scale bar, 50 μm. The bar graph shows the percentage of Tuj1– and GFP^+^ had increased in the CP of *Mettl3* KD mice (**F**) (*n* = 3). Data are presented as mean ± S.E.M., unpaired *t*-test, ^*^*P* < 0.05; ^**^*P* < 0.01; ^**^**P* < 0.001. Representative images showing the change of dendritic numbers and length in *Mettl3* KD, and *Mettl3* overexpression cells (G, H). Representative immunofluorescence images of hippocampal neurons transfected with control and *Mettl3* KD plasmids, respectively (**I**). Scale bar, 50 μm. shRNA, short hairpin RNA.

Supplementary Figure S5 **Effects of *Mettl3* knockdown on m^6^A distribution and gene expression** Venn diagram illustrating the overlap of m^6^A peaks detected from two independent MeRIP-seq datasets of different samples (**A**). Scatter plots showing gene expression between two independent biological replicates of different samples. Pearson correlation coefficients are shown (**B**). RPKM numbers showed the differential expression of several selected genes in proliferation and differentiation conditions (**C**). RPKM numbers of several selected genes which showed differential expressions in proliferating *Mettl3* KD aNSCs (**D**). GO analysis for non-m^6^A-tagged up- (**E**) and down- (**F**) regulated transcripts.

Supplementary Figure S6 **Validation of m^6^A modification at *Ezh2*, and the effect of *Mettl3* modulation on the expression of *Ezh2*** IGV images showing m^6^A peaks at the *Ezh2* transcript (**A**). The validation of m^6^A peak at the Ezh2 transcript by m^6^A-IP-qPCR (**B**). m^6^A-IP followed by qPCR results showed that *Mettl3* KD had significantly decreased m^6^A density at the *Ezh2* transcript (**C**). qRT-PCR results showed Mettl3 KD did not affect the mRNA level of *Ezh2* (**D**). The overexpression of both wild-type (WT) and mutant (Mut) Ezh2 significantly increased the mRNA level (**E**) and protein level of Ezh2 (**F**), but did not show any observable effect on Mettl3 at the protein level (**F**) in aNSCs. The overexpression of both wild–type (WT) and mutant (Mut) *Ezh2* significantly increased the mRNA levels of Ezh2 (**G**) and protein levels of Ezh2 (**H**), but did not affect the Mettl3 level (**H**) in N2a cells. qRT-PCR results showed that the overexpression of WT or Mut *Ezh2* did not recover the decreased mRNA level of *Mettl3* (**I**) (*n* = 3). Western blots results showed that the overexpression of Mettl3 significantly increased Ezh2 (**J**) and H3K27me3 (**K**) levels in N2a cells (*n* = 3). RNA dot-blot results showed that the overexpression of *Mettl3* had significantly increased m^6^A levels (**L**) (*n* = 3). A schematic model illustrating how *Mettl3* deficiency altered gene expression, especially *Ezh2* and led to abnormal neurogenesis and neuronal development (M
