# Supplementary material for: m6A Regulates Neurogenesis and Neuronal Development by Modulating Histone Methyltransferase Ezh2
Source: Genomics Proteomics Bioinformatics. 2019 May 30;17(2):154–68. doi: 10.1016/j.gpb.2018.12.007 (PMC6620265; doi:10.1016/j.gpb.2018.12.007)
Supplement: Supplementary Figure S1 — Validation of the homogeneity, self-renewal capability and multipotency of aNSCs Representative immunofluorescence staining showing that the cultured aNSCs were positive for NSC markers Nestin and Sox2 (A). BrdU staining revealed the self-renewal capability of Nestin+ (B) and Sox2+ (C) aNSCs. Upon differentiation, aNSCs generated neuronal cells (Tuj1+) and astrocytes (GFAP+) (D). qRT-PCR analysis of mRNA levels of multiple cell markers Nestin (NSC marker), Tuj1 and NeuroD (neuronal markers), Gfap and S100β (glial markers) in proliferation and differentiation conditions of aNSCs (E) (n = 3). Actin mRNA was used as an internal control. Data are presented as mean ± S.E.M., unpaired t-test, *P < 0.05; **P < 0.01; ***P < 0.001. Scale bar, 50 μm. [file mmc1.pptx]

## Slide 1
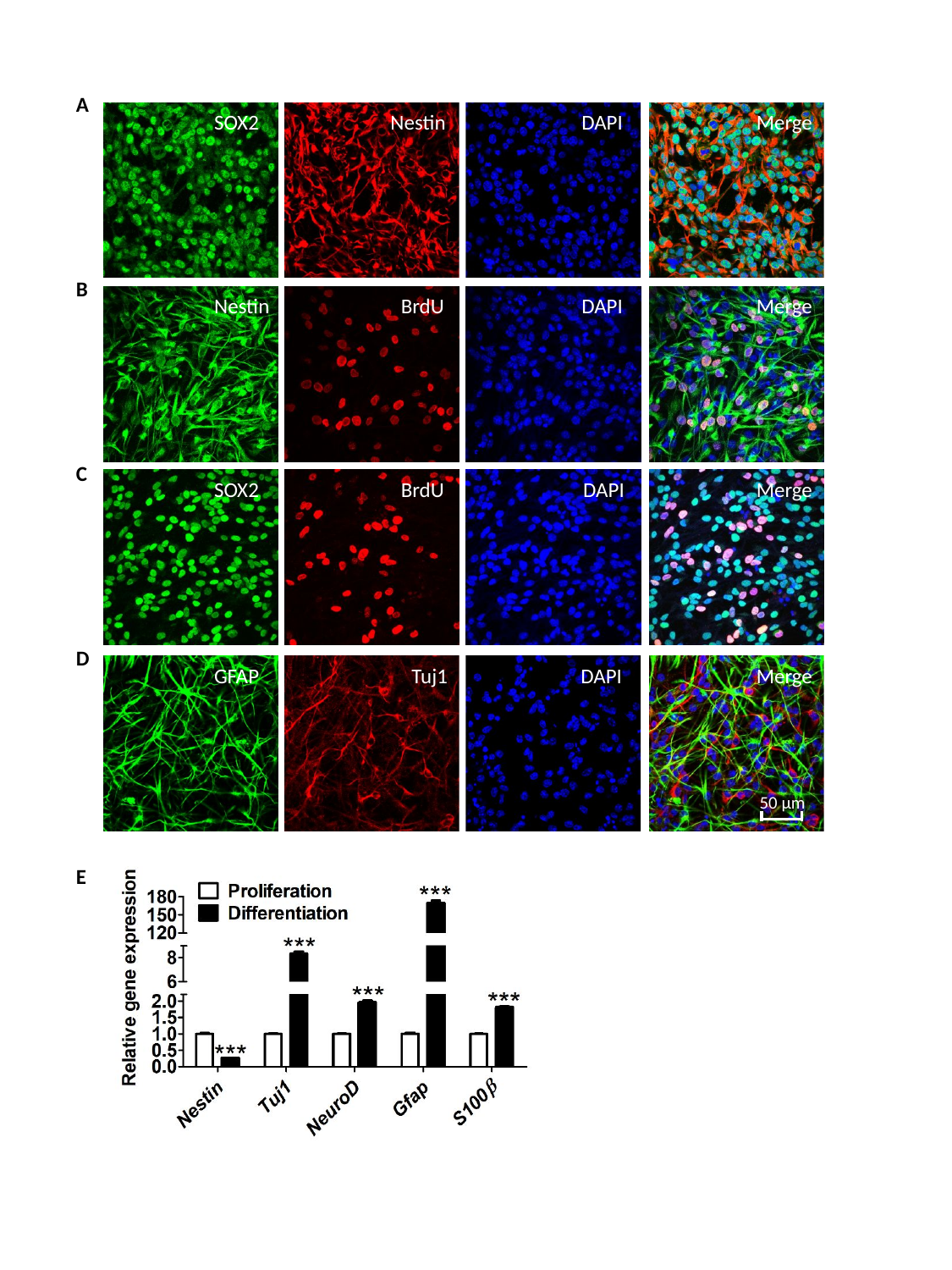

A
SOX2
Nestin
DAPI
Merge
B
Nestin
BrdU
DAPI
Merge
C
SOX2
BrdU
DAPI
Merge
D
GFAP
Tuj1
DAPI
Merge
50 µm
E
