# Supplementary material for: m6A Regulates Neurogenesis and Neuronal Development by Modulating Histone Methyltransferase Ezh2
Source: Genomics Proteomics Bioinformatics. 2019 May 30;17(2):154–68. doi: 10.1016/j.gpb.2018.12.007 (PMC6620265; doi:10.1016/j.gpb.2018.12.007)
Supplement: Supplementary Figure S2 — Expression of m6A modification-related multiple genes in cultured aNSCs and detection of m6A in brain Representative immunofluorescence staining of Mettl14 with aNSC markers Sox2 (A), Nestin (B), and the neuronal cell marker Tuj1 (C) and astrocyte marker Gfap (D). qRT-PCR analysis of Mettl3 and Mettl14 mRNA levels between the proliferation and differentiation of aNSCs, respectively (E). The comparison of mRNA levels of Wtap, Fto and Alkbh5 between the proliferation and differentiation of aNSCs (F). Representative immunofluorescence staining showing that m6A was enriched in mature neurons (NeuN+) in the hippocampus of postnatal day 14 (P14) mouse brains (G). RNase treatment significantly depleted the signal intensity of m6A immunofluorescence staining (H). Scale bar through A–D and G, H, 50 μm. [file mmc2.pptx]

## Slide 1
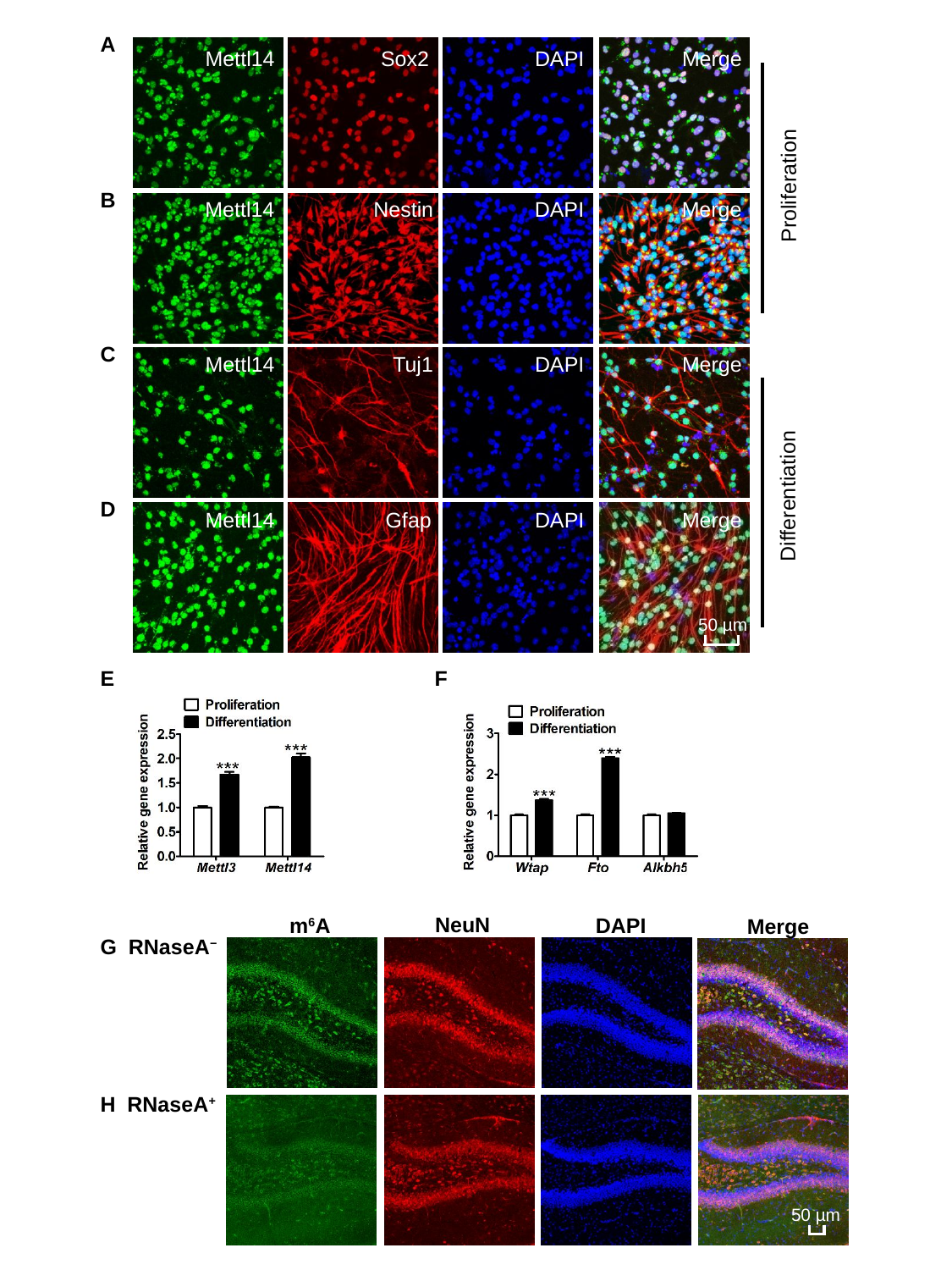

A
Mettl14
DAPI
Merge
Sox2
Proliferation
B
Mettl14
Nestin
DAPI
Merge
C
Mettl14
Tuj1
DAPI
Merge
Differentiation
D
Mettl14
Gfap
DAPI
Merge
50 µm
E
F
NeuN
m6A
DAPI
Merge
G RNaseA−
H RNaseA+
50 µm
