# Supplementary material for: m6A Regulates Neurogenesis and Neuronal Development by Modulating Histone Methyltransferase Ezh2
Source: Genomics Proteomics Bioinformatics. 2019 May 30;17(2):154–68. doi: 10.1016/j.gpb.2018.12.007 (PMC6620265; doi:10.1016/j.gpb.2018.12.007)
Supplement: Supplementary Figure S3 — Effects of Mettl3 knockdown on the homogeneity and proliferation of aNSCs Representative immunofluorescence staining of NSC marker Sox2 in control and Mettl3 KD aNSCs (A). Representative immunofluorescence staining of NSC marker Nestin in control and Mettl3 KD aNSCs (B). Representative immunofluorescence staining of cell cycle marker Ki67 in control and Mettl3 KD aNSCs (C). Quantification results showed that Mettl3 KD did not affect the percentage of Sox2+ (D), Nestin+ (E) and Ki67+ (F) cells compared to control groups, respectively (n = 3). Quantitative analysis showed Mettl3 KD induced the decrease of phosphor-histone H3 positive (p-H3+) cells (G) (n = 3). Data are presented as mean ± S.E.M., unpaired t-test, *P < 0.05; **P < 0.01; ***P < 0.001. Scale bar, 50 μm. [file mmc3.pptx]

## Slide 1
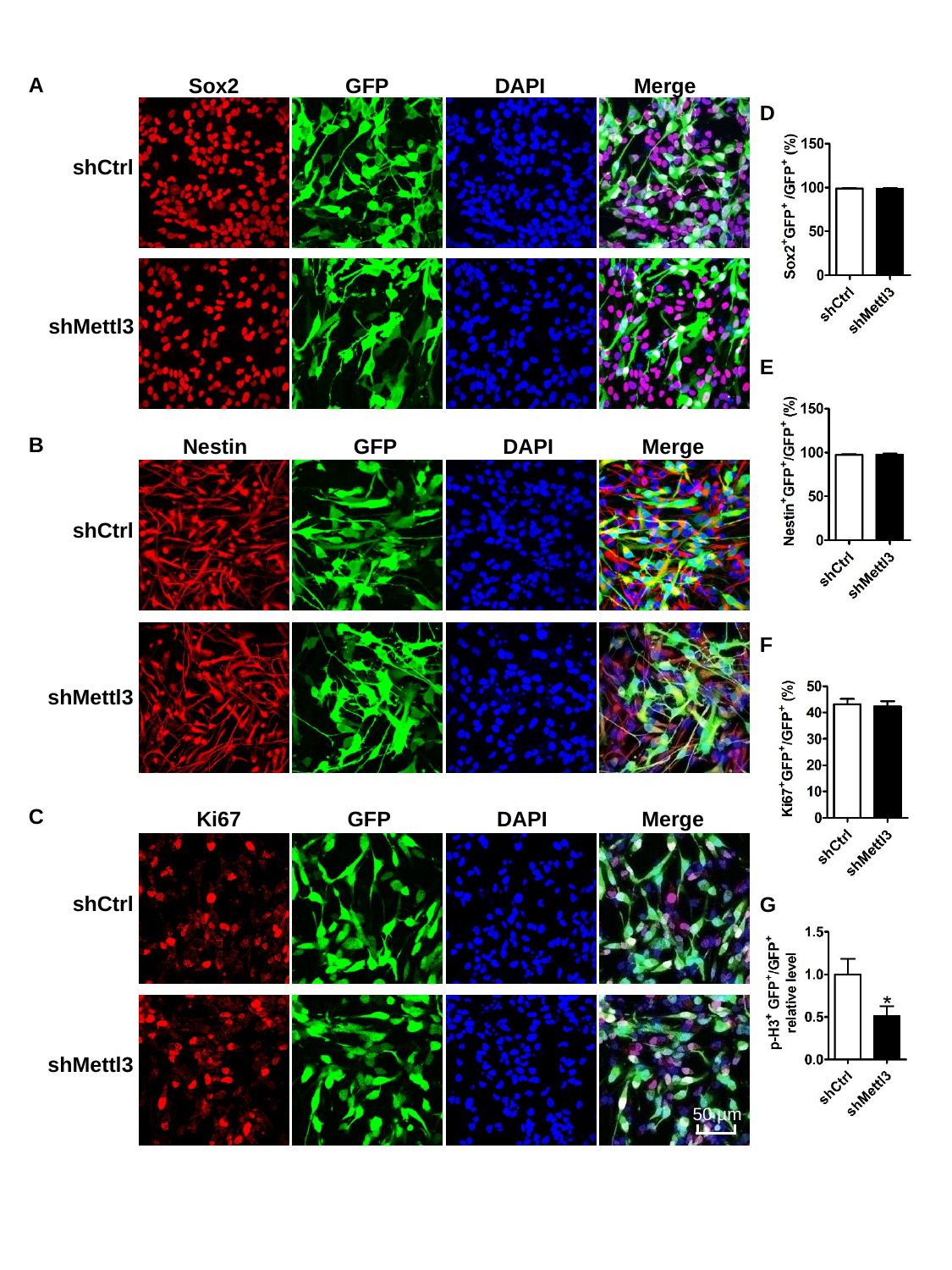

A
Sox2 GFP DAPI Merge
D
shCtrl
shMettl3
E
B
Nestin GFP DAPI Merge
shCtrl
F
shMettl3
C
Ki67 GFP DAPI Merge
shCtrl
G
shMettl3
50 µm
