# Supplementary material for: m6A Regulates Neurogenesis and Neuronal Development by Modulating Histone Methyltransferase Ezh2
Source: Genomics Proteomics Bioinformatics. 2019 May 30;17(2):154–68. doi: 10.1016/j.gpb.2018.12.007 (PMC6620265; doi:10.1016/j.gpb.2018.12.007)
Supplement: Supplementary Figure S4 — Effects of Mettl3 knockdown and overexpression on the differentiation of aNSCs and neuronal development qRT-PCR results showed the mRNA levels of Tuj1, NeuroD, Gfap and S100β in the control and Mettl3 KD samples (differentiation conditions) (A) (n = 3). qRT-PCR results showed the mRNA levels of Mettl3, Tuj1, NeuroD, Gfap and S100β in the control and Mettl3 over-expression samples (differentiation conditions) (B) (n = 3). Luciferase assay results show that the overexpression of Mettl3 had increased the promoter activities of the pan-neuronal marker, NeuroD (C), but decreased the promoter activities of glial cell marker, Gfap (D) (n = 3). Representative immunofluorescence images of in utero electroporation (E). The control or Mettl3-shRNA plasmid was electroporated into E13.5 embryonic mouse brains, sacrificed at E14.5, and the embryonic brains were sectioned with Cryostat. The harvested sections underwent immunofluorescence staining with images taken using an Olympus confocal microscope (at least five sections from each brain, 2 pups from each litter and 2 litters were used each group). Scale bar, 50 μm. The bar graph shows the percentage of Tuj1– and GFP+ had increased in the CP of Mettl3 KD mice (F) (n = 3). Data are presented as mean ± S.E.M., unpaired t-test, *P < 0.05; **P < 0.01; ***P < 0.001. Representative images showing the change of dendritic numbers and length in Mettl3 KD, and Mettl3 overexpression cells (G, H). Representative immunofluorescence images of hippocampal neurons transfected with control and Mettl3 KD plasmids, respectively (I). Scale bar, 50 μm. shRNA, short hairpin RNA. [file mmc4.pptx]

## Slide 1
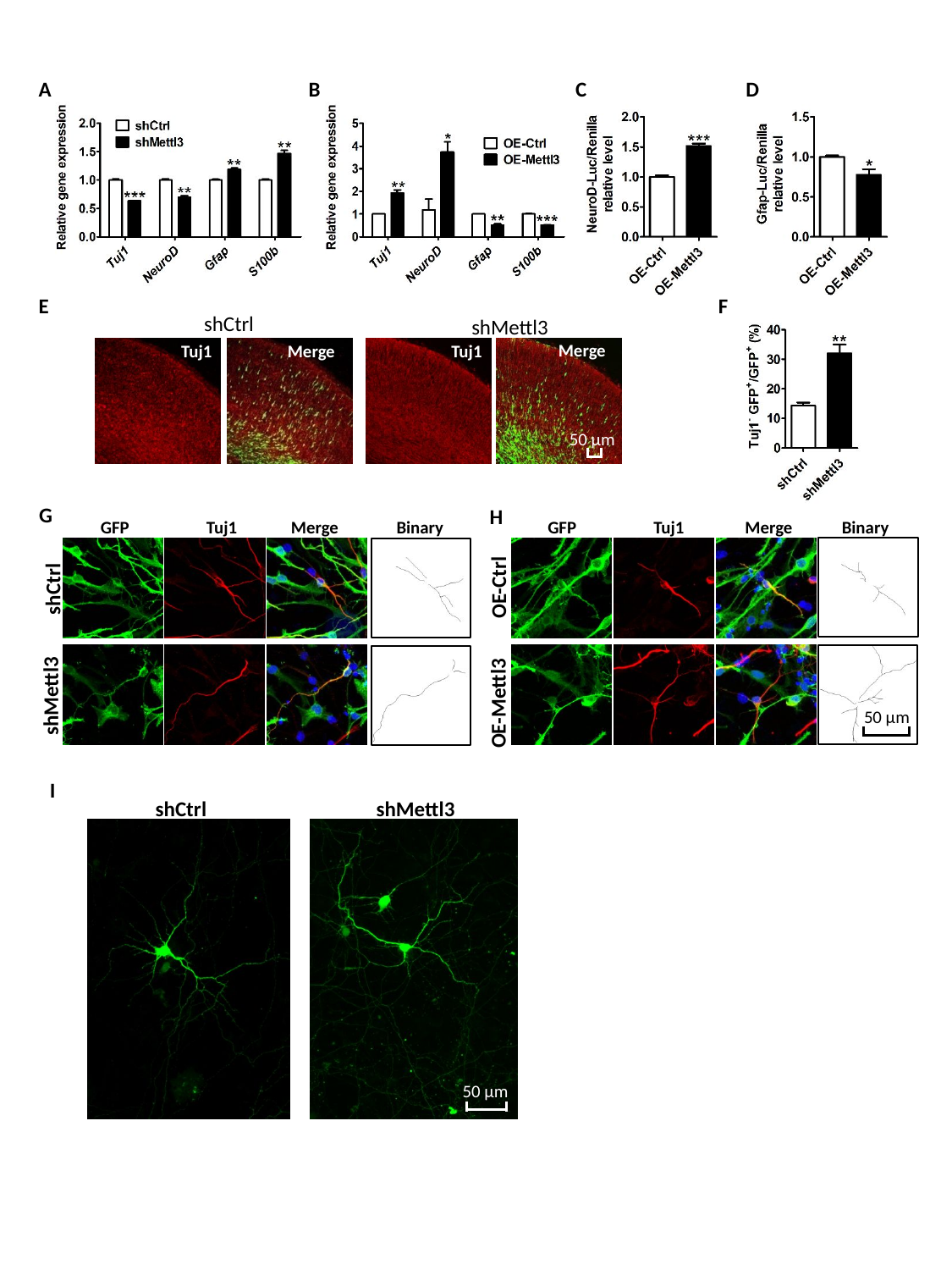

A
B
C
D
E
F
shCtrl
shMettl3
Merge
Tuj1
Merge
Tuj1
50 µm
G
H
GFP
Tuj1
Merge
Binary
GFP
Tuj1
Merge
Binary
OE-Ctrl
shCtrl
OE-Mettl3
shMettl3
50 µm
I
 shCtrl shMettl3
50 µm
