# Supplementary material for: m6A Regulates Neurogenesis and Neuronal Development by Modulating Histone Methyltransferase Ezh2
Source: Genomics Proteomics Bioinformatics. 2019 May 30;17(2):154–68. doi: 10.1016/j.gpb.2018.12.007 (PMC6620265; doi:10.1016/j.gpb.2018.12.007)
Supplement: Supplementary Figure S5 — Effects of Mettl3 knockdown on m6A distribution and gene expression Venn diagram illustrating the overlap of m6A peaks detected from two independent MeRIP-seq datasets of different samples (A). Scatter plots showing gene expression between two independent biological replicates of different samples. Pearson correlation coefficients are shown (B). RPKM numbers showed the differential expression of several selected genes in proliferation and differentiation conditions (C). RPKM numbers of several selected genes which showed differential expressions in proliferating Mettl3 KD aNSCs (D). GO analysis for non-m6A-tagged up- (E) and down- (F) regulated transcripts. [file mmc5.pptx]

## Slide 1
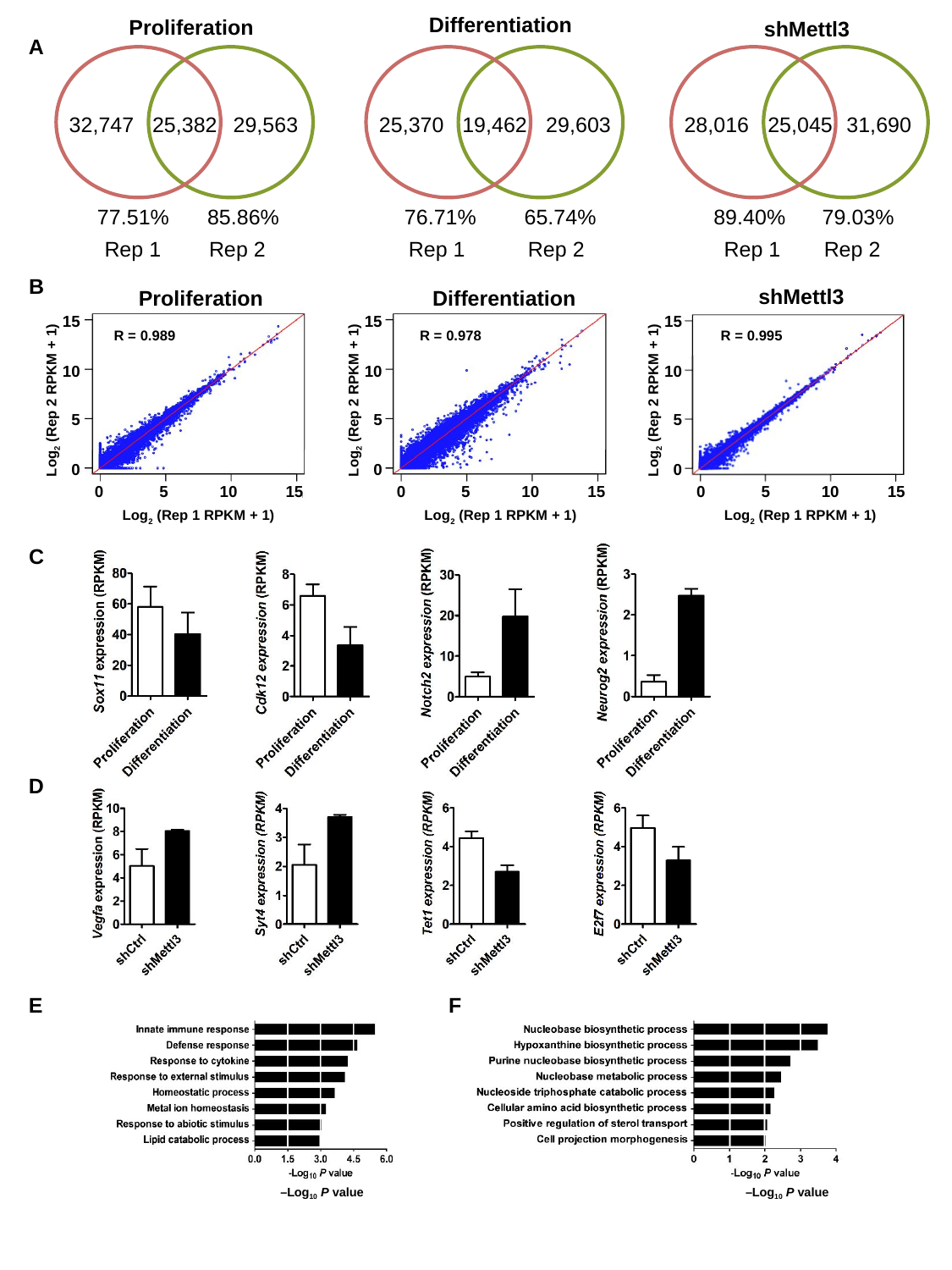

Differentiation
25,370
19,462
29,603
76.71%
65.74%
Proliferation
32,747
25,382
29,563
77.51%
85.86%
shMettl3
A
28,016
25,045
31,690
89.40%
79.03%
Rep 1
Rep 2
Rep 1
Rep 2
Rep 1
Rep 2
B
shMettl3
Proliferation
Differentiation
15
10
Log2 (Rep 2 RPKM + 1)
5
0
0
5
10
15
Log2 (Rep 1 RPKM + 1)
15
10
Log2 (Rep 2 RPKM + 1)
5
0
0
5
10
15
Log2 (Rep 1 RPKM + 1)
15
10
Log2 (Rep 2 RPKM + 1)
5
0
0
5
10
15
Log2 (Rep 1 RPKM + 1)
R = 0.978
R = 0.995
R = 0.989
C
D
E
F
–Log10 P value
–Log10 P value
