# Supplementary material for: m6A Regulates Neurogenesis and Neuronal Development by Modulating Histone Methyltransferase Ezh2
Source: Genomics Proteomics Bioinformatics. 2019 May 30;17(2):154–68. doi: 10.1016/j.gpb.2018.12.007 (PMC6620265; doi:10.1016/j.gpb.2018.12.007)
Supplement: Supplementary Figure S6 — Validation of m6A modification at Ezh2, and the effect of Mettl3 modulation on the expression of Ezh2 IGV images showing m6A peaks at the Ezh2 transcript (A). The validation of m6A peak at the Ezh2 transcript by m6A-IP-qPCR (B). m6A-IP followed by qPCR results showed that Mettl3 KD had significantly decreased m6A density at the Ezh2 transcript (C). qRT-PCR results showed Mettl3 KD did not affect the mRNA level of Ezh2 (D). The overexpression of both wild-type (WT) and mutant (Mut) Ezh2 significantly increased the mRNA level (E) and protein level of Ezh2 (F), but did not show any observable effect on Mettl3 at the protein level (F) in aNSCs. The overexpression of both wild–type (WT) and mutant (Mut) Ezh2 significantly increased the mRNA levels of Ezh2 (G) and protein levels of Ezh2 (H), but did not affect the Mettl3 level (H) in N2a cells. qRT-PCR results showed that the overexpression of WT or Mut Ezh2 did not recover the decreased mRNA level of Mettl3 (I) (n = 3). Western blots results showed that the overexpression of Mettl3 significantly increased Ezh2 (J) and H3K27me3 (K) levels in N2a cells (n = 3). RNA dot-blot results showed that the overexpression of Mettl3 had significantly increased m6A levels (L) (n = 3). A schematic model illustrating how Mettl3 deficiency altered gene expression, especially Ezh2 and led to abnormal neurogenesis and neuronal development (M [file mmc6.pptx]

## Slide 1
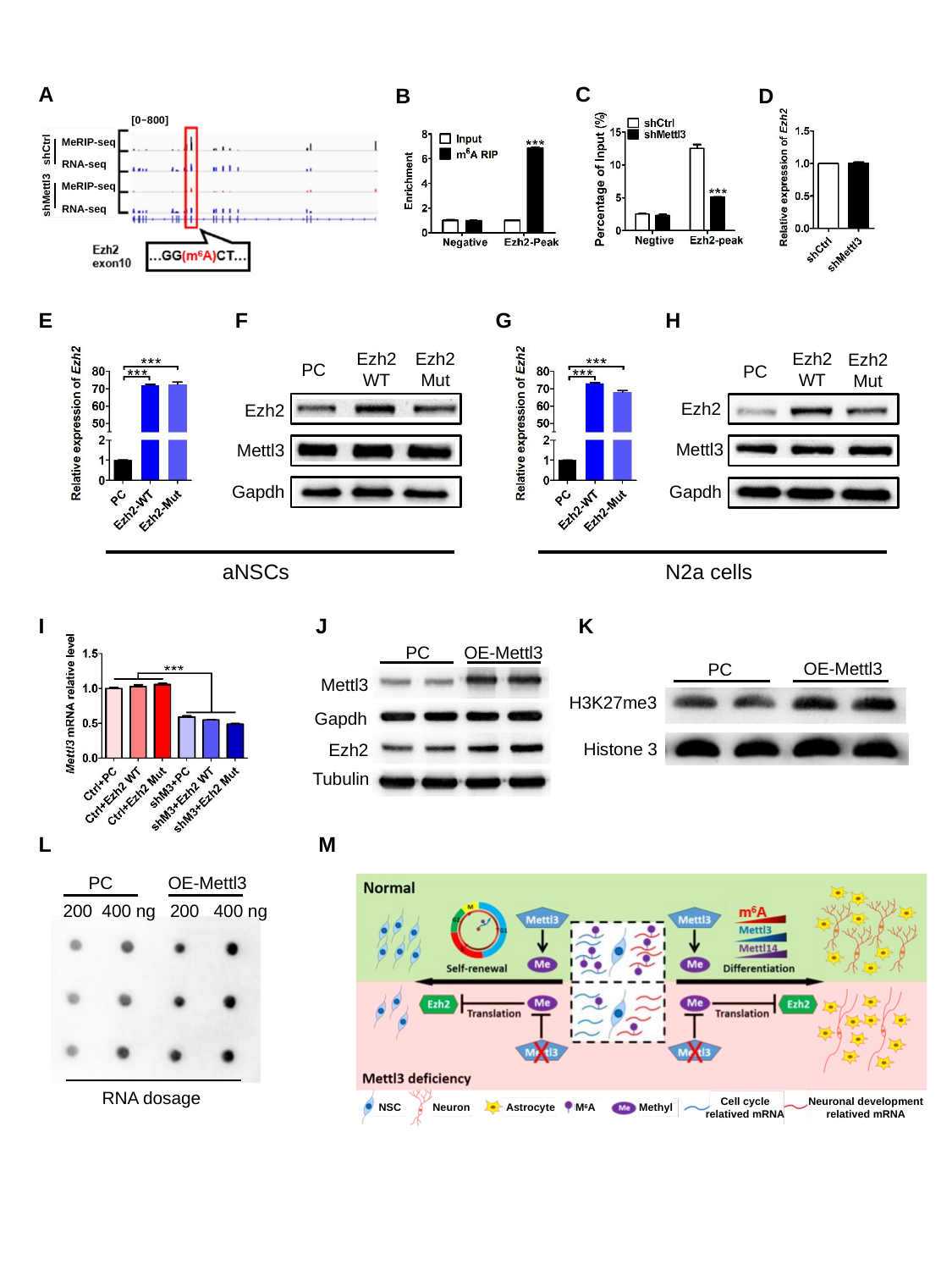

A
C
B
D
[0–800]
MeRIP-seq
shCtrl
RNA-seq
MeRIP-seq
shMettl3
RNA-seq
E
F
G
H
Ezh2
WT
Ezh2
Mut
Ezh2
WT
Ezh2
Mut
PC
PC
Ezh2
Ezh2
Mettl3
Mettl3
Gapdh
Gapdh
aNSCs
N2a cells
I
J
K
 OE-Mettl3
PC
Mettl3
Gapdh
Ezh2
Tubulin
OE-Mettl3
PC
H3K27me3
Histone 3
L
M
OE-Mettl3
PC
Cell cycle
relatived mRNA
Neuronal development
relatived mRNA
NSC
Neuron
Astrocyte
M6A
Methyl
200 400 ng 200 400 ng
RNA dosage
